# A Rare Case of Fatal Endobronchial Mucormycosis Masquerading as Endobronchial Tuberculosis

**DOI:** 10.3390/medicina56020064

**Published:** 2020-02-06

**Authors:** Minjeong Kim, Jun Hyeok Lim, Mihwa Park, Hyung Keun Cha, Lucia Kim, Hae-Seong Nam

**Affiliations:** 1 Division of Pulmonology, Department of Internal Medicine, Inha University Hospital, Inha University School of Medicine, Incheon 22332, Korea; aube1800@naver.com (M.K.); tboy1012@naver.com (J.H.L.); bami.park@gmail.com (M.P.); silmiboy@naver.com (H.K.C.); 2 Department of Pathology, Inha University Hospital, Inha University School of Medicine, Incheon 22332, Korea; luciado@inha.ac.kr

**Keywords:** endobronchial mucormycosis, endobronchial tuberculosis, fungal infection, diabetes mellitus

## Abstract

Pulmonary mucormycosis is a relatively rare but often fatal opportunistic fungal infection that occurs mostly in immunocompromised patients. Endobronchial mucormycosis, a distinct clinical form of pulmonary mucormycosis, is very rare, and only a few cases have been reported. The most common bronchoscopic findings in patients with endobronchial mucormycosis are stenosis, erythematous mucosa and airway obstruction. Here, we present a case of fatal endobronchial mucormycosis mimicking actively caseating endobronchial tuberculosis in a young diabetic patient living in a country with an intermediate tuberculosis burden.

## 1. Introduction

Pulmonary mucormycosis (PM) is an opportunistic fungal infection that occurs after the inhalation of spores into the bronchioles and alveoli. While relatively uncommon, its incidence is increasing. The disease progresses rapidly and is often fatal, with an overall mortality rate as high as 76% [1]. The clinical features of PM are nonspecific and are not easily distinguished from those of other pulmonary infections, such as bacterial, mycobacterial and other fungal infections [1,2,3]. PM is generally classified into two clinical forms. The more common form produces parenchymal disease with a rapidly progressive clinical course, whereas the endobronchial form is very rare and mainly involves the large airways. Endobronchial mucormycosis (EM) has been reported in only a few cases. Its bronchoscopic findings include stenosis, erythematous mucosa and airway obstruction [3,4,5,6,7]. Here we report a case of fatal EM mimicking actively caseating endobronchial tuberculosis (EBTB) in a young diabetic patient.

## 2. Case Report

**Figure 1:**
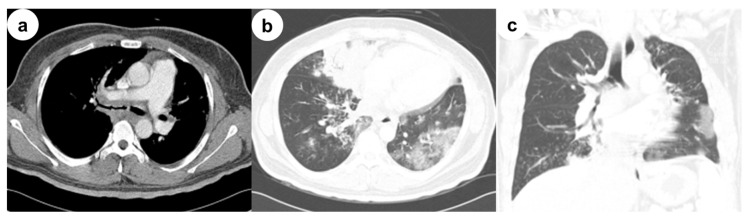
Initial contrast chest computed tomography (CT). (**a**) Selected axial image of the soft-tissue window on chest CT shows the thickening of the right main bronchus, a peribronchial soft-tissue lesion and lymphadenopathy; (**b**) Selected axial and (**c**) coronal images of the lung window on chest CT shows consolidation in the right middle lobe, diffuse and patchy densities with focal consolidations involving both lower lobes, and thickening of the right main and intermedius bronchi.

**Figure 2:**
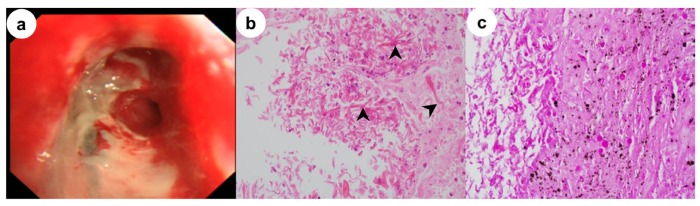
(**a**) Bronchoscopy images show a whitish cheese-like material on the right upper bronchus and swollen and hyperemic mucosae; A bronchoscopic biopsy specimen shows (**b**) extensive necrosis, nonseptate hyphae with irregular, right-angle branching (arrowhead) and branches of different thickness on the hematoxylin- and eosin-stained sections (x400); (**c**) Extensive necrosis and fungal hyphae are revealed by periodic acid Schiff staining (x400).

A 32-year-old man diagnosed with type I diabetes mellitus (DM) 17 years previously presented with a fever, productive cough and dyspnea lasting a few days. He had no history of smoking, alcohol consumption or intravenous drug abuse. On admission, physical examination revealed a respiratory rate of 22 beats/min, temperature of 37.9 °C, pulse of 110 beats/min, and blood pressure of 130/80 mmHg. Arterial blood gas analysis revealed a partial oxygen pressure of 61 mmHg, partial carbon dioxide pressure of 37 mmHg, bicarbonate level of 26 mEg/L, pH of 7.46 and oxygen saturation of 92% at rest in room air. Laboratory data included a white cell count of 15,160/mm^3^ with 77% neutrophils, a C-reactive protein level of 8.42 mg/dL (normal, 0–0.3 mg/dL), a serum glucose level of 86 mg/dL, and a serum creatinine level of 1.53 mg/dL (normal, 0.4–1.5 mg/dl). However, his glycated hemoglobin (HbA1c) level was 14.9%. Auscultation revealed inspiratory crackles in both lung bases. Empirical treatment with ceftriaxone and azithromycin was initiated to lower the fever. Chest computed tomography (CT) showed consolidation in the right middle lobe, diffuse and patchy densities with focal consolidations involving both lower lobes, thickening of the right main and intermedius bronchi, and multiple mediastinal lymphadenopathies (Figure 1). Bronchoscopy showed that the right upper bronchus was covered with whitish cheese-like material, and the mucosae were swollen and hyperemic, leading to the narrowing of the lumen to the right intermedius bronchus (Figure 2). Based on the chest CT and bronchoscopy findings, the patient was diagnosed with EBTB and given anti-tuberculosis medication. However, bronchoscopic biopsy demonstrated extensive necrosis and non-septate hyphae with irregular and right-angle branching, indicative of mucormycosis (Figure 2). A diagnosis of EM was made, and treatment consisting of intravenous liposomal amphotericin B was initiated two days after bronchoscopy. Bronchial washings for mycobacterial culture and cytology for malignant cells were negative. The patient refused additional interventional bronchoscopy or surgical debridement. Although treatment with the antifungal agent resulted in a temporary improvement in the patient’s symptoms, he developed respiratory failure 30 days after the antifungal treatment and required intubation and mechanical ventilation. The patient died from massive hemoptysis 3 days after intubation. This study was approved by the Institutional Review Board of Inha University Hospital (INHAUH 202002003, approved in January 2020). Verbal informed consent was obtained from the patient’s mother by telephone for publication of this manuscript and any accompanying images.

## 3. Discussion

Mucormycosis is an emerging angioinvasive infection caused by ubiquitous filamentous fungi in the order Mucorales (class: Zygomycetes). Although the disease is uncommon, it is the third most common fungal infection after candidiasis and aspergillosis. Mucormycosis occurs mostly in immunocompromised patients, such as those with hematologic malignancies with or without stem cell transplantation, severe neutropenia, poorly controlled DM with or without diabetic ketoacidosis, iron overload, or prolonged use of corticosteroids [1,3,8]. Invasive mucormycosis has a high mortality rate. Six major clinical forms are recognized based on the clinical presentation and anatomic site—the rhinocerebral, pulmonary, cutaneous, gastrointestinal, disseminated, and uncommon rare (e.g., endocarditis, osteomyelitis, peritonitis, and renal infection) forms. The lungs (24%) are the second most common site of invasive mucormycosis, after the sinuses (39%) [1,8]. 

PM is not easy to differentiate from other pulmonary infections because the clinical manifestations and laboratory findings are nonspecific. The most common forms of PM are parenchymal disease, characterized by diffuse infiltrations in the lung and rapid development of respiratory failure, and endobronchial disease, which predominantly affects the large airways [1,5]. EM is fairly rare, and only a few cases have been reported [3,4,5,6,7]. According to a literature review, EM accounts for 34% of PM cases [4]. Another study found that only 60 cases of EM have been reported since 1980 [7]. The most common bronchoscopic findings of EM are stenosis, erythematous mucosa and airway obstruction [3,4,7]. As demonstrated by this case, EM has a predilection for diabetic patients (66.7%) without ketoacidosis and may be misdiagnosed as tuberculosis, as documented in four cases so far [7]. However, in these cases, actively caseating EBTB lesions, the most typical bronchoscopic finding in EBTB, were diagnosed in bronchial mucosae that were swollen, hyperemic and diffusely covered with a whitish cheese-like material [9]. These findings were very similar to those of our patient, which together with the CT findings and the fact that the patient was young and living in a country with an intermediate tuberculosis burden, led us to suspect EBTB. To our knowledge, EM masquerading as actively caseating EBTB, including in bronchoscopy, has not been reported previously. 

A definite diagnosis of PM requires the histologic demonstration of tissue invasion by fungi with characteristic non-septate hyphae and right-angle branching. Cultures of sputum or histologic specimens are usually negative [1,8]. Amphotericin B is the drug of choice for the antifungal therapy of mucormycosis. As in our patient, lipid formulations are generally administered due to their delivery of a high dose with less nephrotoxicity compared with other formulations [8]. Although recent therapeutic advances have the potential to improve the outcome of mucormycosis, the mortality rate in disseminated mucormycosis remains as high as 96% [1,2,8]. Because the causative fungus in mucormycosis is angioinvasive, aggressive and rapidly growing, early surgical intervention combined with medical therapy should be considered, as this approach seems to result in more favorable outcomes compared with medical treatment alone [2,10]. For patients who are not candidates for surgery or refuse surgical intervention, as did our patient, improved outcomes await the development of new antifungal agents and clinical trials of combinations of agents active against most mucormycoses, such as amphotericin B, posaconazole and isavuconazole. Until more effective treatment becomes available, an aggressive diagnostic approach leading to an early diagnosis, the control or reduction of underlying risk factors, prompt administration of appropriate antifungal therapy and surgical debridement, when applicable, are critical for the successful treatment of patients with mucormycosis. 

## 4. Conclusions

Although fairly rare, EM is a clinically distinct life-threatening form of PM. However, with the growing number of immunocompromised patients, including those with DM, the incidence of EM is increasing. Therefore, a high level of suspicion and an aggressive diagnostic approach are essential for patients presenting with progressive pneumonia despite adequate antibiotic treatment. An awareness of the similar bronchoscopic findings of EM and EBTB, especially in countries with an intermediate or high tuberculosis burden, is critical for the timely diagnosis and adequate treatment of this potentially fatal disease.
